# Formaldehyde Molecules Adsorption on Zn Doped Monolayer MoS_2_: A First-Principles Calculation

**DOI:** 10.3389/fchem.2020.605311

**Published:** 2021-04-16

**Authors:** Huili Li, Ling Fu, Chaozheng He, Jinrong Huo, Houyong Yang, Tingyue Xie, Guozheng Zhao, Guohui Dong

**Affiliations:** ^1^ Key Laboratory of Magnetic Molecules, Magnetic Information Materials Ministry of Education, The School of Chemistry and Material Science, Shanxi Normal University, Linfen, China; ^2^ College of Agricultural Engineering, Nanyang Normal University, Nanyang, China; ^3^ College of Resources and Environmental Engineering, Tianshui Normal University, Tianshui, China; ^4^ Institute of Environmental and Energy Catalysis, School of Materials Science and Chemical Engineering, Xi’an Technological University, Xi’an, China; ^5^ Shaanxi Key Laboratory of Optoelectronic Functional Materials and Devices, School of Materials Science and Chemical Engineering, Xi’an Technological University, Xi’an, China; ^6^ School of Physics and Electronic Science, Shanxi Datong University, Shanxi, China; ^7^ School of Environmental Science and Engineering, Shaanxi University of Science and Technology, Xi’an, China

**Keywords:** first-principles calculation, monolayer MoS_2_, H_2_CO, adsorption energy, gas sensitivity

## Abstract

Based on the first principles of density functional theory, the adsorption behavior of H_2_CO on original monolayer MoS_2_ and Zn doped monolayer MoS_2_ was studied. The results show that the adsorption of H_2_CO on the original monolayer MoS_2_ is very weak, and the electronic structure of the substrate changes little after adsorption. A new kind of surface single cluster catalyst was formed after Zn doped monolayer MoS_2_, where the ZnMo_3_ small clusters made the surface have high selectivity. The adsorption behavior of H_2_CO on Zn doped monolayer MoS_2_ can be divided into two situations. When the H-end of H_2_CO molecule in the adsorption structure is downward, the adsorption energy is only 0.11 and 0.15 eV and the electronic structure of adsorbed substrate changes smaller. When the O-end of H_2_CO molecule is downward, the interaction between H_2_CO and the doped MoS_2_ is strong leading to the chemical adsorption with the adsorption energy of 0.80 and 0.98 eV. For the O-end-down structure, the adsorption obviously introduces new impurity states into the band gap or results in the redistribution of the original impurity states. All of these may lead to the change of the chemical properties of the doped MoS_2_ monolayer, which can be used to detect the adsorbed H_2_CO molecules. The results show that the introduction of appropriate dopant may be a feasible method to improve the performance of MoS_2_ gas sensor.

## Introduction

In recent years, two-dimensional (2D) materials have attracted much attention due to their unique physical, chemical and electrical properties (Abbasi and Sardroodi, 2018a; Abbasi and Sardroodi, 2018b; Wu et al., 2018; Abbasi, 2019; Abbasi and Sardroodi, 2019). Among them, monolayer MoS_2_ belongs to hexagonal system, which is a typical 2D layered transition metal sulfide with sheet structure similar to graphene (Pan et al., 2020). It has been studied due to the excellent electronic structure, chemical and thermal stability, high surface activity and high strength (Xu et al., 2013). Compared with the zero gap of graphene, single-layer MoS_2_ has considerable direct band gap, which is suitable for light emitter, energy conversion and solar cell (Yu et al., 2016). Meanwhile, MoS_2_ has large specific surface area, surface activity and excellent adsorption capacity, so it is a special gas storage material or gas sensing material source (Zhang et al., 2017; Zhang et al., 2018; Zhou et al., 2018; Chen et al., 2019; Kathiravan et al., 2019; Tan et al., 2020). These excellent properties make the monolayer MoS_2_ have potential applications in the field of gas sensing.

However, due to the lack of free bonds on the surface of intact MoS_2_, which is chemically inert (Wang et al., 2013), the interaction with most gas molecules is limited to physical adsorption (Wanno and Tabtimsai, 2014), resulting in weak interaction between the adsorbent and monolayer MoS_2_, and the change of electronic properties is not obvious, and the original MoS_2_ cannot detect H_2_CO gas molecule (Ma et al., 2016a). Therefore, it is an effective and feasible method to adjust the electronic structure, chemical activity and sensitivity of MoS_2_ by introducing appropriate dopants into defect sites (Ma et al., 2016c; Sharma et al., 2018; Cui et al., 2019; Guo et al., 2019; Ren et al., 2019; Zhang et al., 2019; Guo et al., 2020; Zheng et al., 2020). For example, Lolla et al. have shown that Fe and Co. doped monolayer MoS_2_ can enhance the adsorption of O due to the partial occupation of *d*-orbitals on Fermi level, and the introduction of doping significantly improves the catalytic activity of MoS_2_ monolayer (Lolla and Luo, 2020). (Abbas et al., 2018) proposed that compared with the original monolayer MoS_2_, N and P doped atoms can enhance the sensing sensitivity of monolayer MoS_2_ to O_2_ and NO gas molecules (Abbas et al., 2018). (Sahoo et al., 2016) have shown that the original monolayer MoS_2_ is inert to gas molecules such as NH_3_ and NO_2_, and the detection sensitivity of antisite doped with MoS_2_ for these gas molecules and other chemical substances is significantly improved (Sahoo et al., 2016). Luo et al. have shown that Al, Si and P doped atoms increase the orbital hybridization effect between NO_2_, NH_3_ molecules and monolayer MoS_2_, and promote the electron transfer. The doped monolayer MoS_2_ has better adsorption performance than the undoped monolayer MoS_2_ (Luo et al., 2016). Ma et al. have shown that when Au, Fe, CO and Ni are doped into the monolayer MoS_2_, the charge transfer occurs, the distance between the adsorbed molecule and the dopant is shortened, the adsorption energy is increased, and the gas sensitivity to H_2_, CO, NO and O_2_ is increased (Ma et al., 2016b; Kwak et al., 2018; Kwon et al., 2018; Wang et al., 2019a). Au doped monolayer MoS_2_ has high charge transfer and strong orbital hybridization ability. Doping Au atoms affect the electronic structure of MoS_2_ monolayer, thus improving the adsorption capacity, so that the adsorption structure of C_2_H_6_ and C_2_H_4_ molecules on Au doped MoS_2_ monolayer is relatively stable (Qian et al., 2020). In conclusion, although there are some reports on the surface activity of doped monolayer MoS_2_, the adsorption of formaldehyde on the surface of transition metal Zn doped MoS_2_ has not been confirmed. Therefore, the geometry, electronic structure and small molecule adsorption of different transition metal atom doped monolayer MoS_2_ system can be obtained by theoretical simulation method, which has guiding significance for the study of the unique gas sensing properties, adsorption properties and chemical activities of transition metal atom doped monolayer MoS_2_.

At present, toxic gas molecules are one of the main problems of environmental pollution. H_2_CO is a common toxic gas. H_2_CO is widely used in household materials (Chung et al., 2013; Salthammer, 2013). Long term exposure to H_2_CO will irritate the eyes and throat, make breathing difficult, and even pose a serious threat to the lungs. Therefore, it is of great significance to detect and control H_2_CO in home, residence and scientific research (Lara-Ibeas et al., 2020; Song et al., 2020; Zhou et al., 2020). Based on the first principle calculation, this paper studies the changes of the configuration, geometric stability and electronic structure of MoS_2_ substrate caused by the adsorption of H_2_CO on the original and Zn doped monolayer MoS_2_ gas after the S vacancy is filled with Zn dopant, and the adsorption energy, adsorption structure and charge transfer of gas molecules are analyzed. This study is helpful to find suitable chemical modification methods to improve the performance of MoS_2_ based gas sensors, and has important scientific significance and application prospects for the design of high-efficiency gas sensing materials.

## Computing Method

All calculations were carried out using the first principle method, which was carried out by Vienna *Ab-initio* Simulation Package (VASP) and projector enhanced wave (PAW) method of DFT (Qian et al., 2019; Wang et al., 2019b). The configuration of transition metal Zn doped MoS_2_ monolayer was optimized. The generalized gradient approximation (GGA) and perdew-Burke-Ernzerhof (PBE) are used to calculate the exchange correlation energy. The C *2s2p*, Zn *3d4s* and O *2s2p* states are regarded as valence electrons. In the process of geometric optimization, all internal coordinates are allowed to relax under a fixed lattice constant, and the energy cutoff for plane waves is set to 450 eV. The Brillouin domain integral uses 3 × 3 × 1 (Monkhorst and Pack, 1976) monkhorst pack (MP) grid and 0.1 eV Gaussian smear. The convergence criterion is 10^−5^ eV. When the force applied to the atom is less than 0.01 ev/Å, the optimized structure is obtained. The calculated lattice constant of MoS_2_ is 3.18 Å, which is well consistent with the experimental and theoretical value of 3.20 and 3.18 Å (Ataca and Ciraci, 2011; Le et al., 2014). Dispersive interactions are not included because we are concerned about the effects of chemical bonds and gas molecules on the electronic and magnetic properties of MoS_2_. This method has been used to study gas adsorption on atom doped MoS_2_ (Ma et al., 2016a). MoS_2_ cell is constructed as a 4 × 4 supercell, in which one S atom is replaced by a Zn doped atom, with a total of 48 atoms, including 16 Mo atoms, 31 S atoms and one Zn atom. In order to avoid the interaction between the MoS_2_ monolayer and its periodic image, a vacuum space of 18 Å was added perpendicular to the MoS_2_ layer. The binding energy (*E*_b_) between metal atom and support is defined as *E*_b_ = *E*_tot_ (MoS_2_-S) + *E*_tot_(M) − *E*_tot_ (M + MoS_2_), where *E*_tot_ (M + MoS_2_), *E*_tot_ (MoS_2_-S) and *E*_tot_ (M) are the total energy of M/MoS_2_ catalyst, energy of MoS_2_ vacancy S-based carrier and the energy of single metal atom, respectively. Positive values indicate that the reaction gives off heat. In addition, the adsorption energy (*E*_ads_) was calculated to describe the interaction strength between gas and gas/MoS_2_ catalyst. According to the formula *E*_ads_ = *E*_tot_ (M + MoS_2_) + *E*_tot_ (gas) − *E*_tot_ (gas-M/MoS_2_), *E*_tot_ (M + MoS_2_), *E*_tot_ (gas) and *E*_tot_ (gas-M/MoS_2_) are the energy of M/MoS_2_ catalyst, the energy of gas and the total energy of adsorption system respectively. According to this definition, positive adsorption energy represents exothermic adsorption. The density of states (DOS) is calculated by using the *K* point 5 × 5 × 1 with higher density. The results of DOS were analyzed by P4vasp. Bader charge (Henkelman et al., 2006) was used to analyze the charge transfer. The 3D visualization program Vesta (Momma and Izumi, 2011) was used to visualize all molecular structures, and the electron density difference was obtained to analyze the electron transfer direction. The electron density difference is defined as △*ρ* = *ρ*_gas-M/MoS2_ − *ρ*_M/MoS2_ − *ρ*_gas_, where *ρ*_gas-M/MoS2_, *ρ*_M/MoS2_ and *ρ*_gas_ represent the electron density of adsorption system, M/MoS_2_ catalyst and gas, respectively. The PAW results based on VASP processing and the COHP diagram of LOBSTER 4.0.0 (Local Orbital Basis Suite Towards Electronic-Structure Reconstruction) (Maintz et al., 2016) were used to analyze the bonding.

## Results and Discussion

### Properties of Zn Doped Monolayer MoS_2_ (Zn-MoS_2_)

**FIGURE 1:**
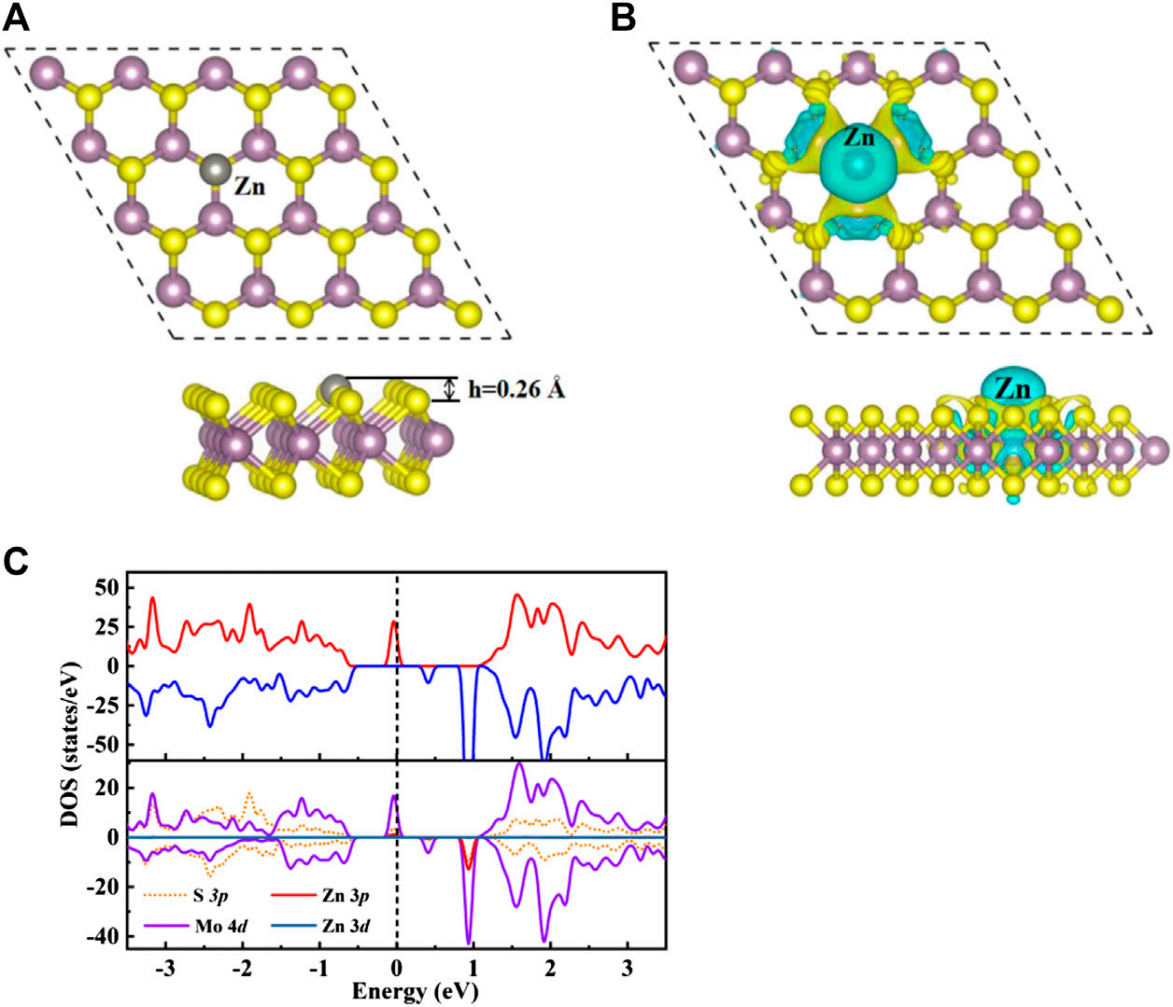
**(A)** Optimize the top view **(top)** and side view **(bottom)** structure of Zn-MoS_2_. Zn atoms are silver spheres. **(B)** The charge density difference diagram of Zn-MoS_2_. The yellow area indicates charge accumulation and the cyan area indicates charge loss. **(C)** TDOS **(upper panels)** and PDOS **(lower panels)** of Zn-MoS_2_ are projected on 3*p* orbital (red curve) and 3*d* orbital (dark blue curve) of Zn, 4*d* orbital (purple curve) of Mo atom and 3*p* orbit (orange dotted line) of S atom. The positive and negative values of DOS indicate spin up and spin down, respectively. The Fermi level is marked with a black dotted line.

Seen from Supplementary Figure S1A, nine possible adsorption sites were considered for H_2_CO adsorb on the original MoS_2_ surface, where T_S_, T_Mo1_/T_Mo2_, H_1_/H_2_ and B_1_/B_2_/B_3_ represent the top of S and Mo atoms, hexagonal ring center and bridge sites. The T_SV_ represent the top site of Zn doped the S vacancy. As depicted in Supplementary Figure S1B, the band structure and total density of states (TDOS) of the original monolayer MoS_2_ show that the original MoS_2_ monolayer is a non-magnetic semiconductor with a direct band gap of 1.74 eV, which is well consistent with the results reported in the literature value of 1.74 eV (Dimple et al., 2017). The calculated binding energy of Zn doping on MoS_2_ surface is 0.5 eV, which indicates that this structure is easy to form under thermodynamic equilibrium conditions due to the exothermic process. As shown in Figure 1B, the charge accumulation and loss can be observed in both Zn and Mo atoms, which means that chemical bonds are formed between Zn and Mo atoms in the ZnMo_3_ clusters, which can be used as a new surface single cluster catalyst (SCC) (Ma et al., 2018) to study the adsorption performance of H_2_CO molecules.

For the average bond length between Zn dopant and adjacent Mo atoms, the calculated average bond length of Zn-Mo is 2.65 Å, which is larger than that of S-Mo bond in original monolayer MoS_2_ with the value of 2.41 Å. As exhibited in Figure 1A, accordingly the expansion of Zn-Mo bond relative to S-Mo bond makes the doped Zn atom protrude 0.26 Å above the S-plane. The electron transfer between dopant and MoS_2_ is calculated by Bader charge analysis. The trend of charge transfer is consistent with that of element electronegativity (Allred, 1961). The paulin electronegativity of Mo is 2.16, which is greater than that of Zn (1.65). Accordingly, Zn dopant loses electrons and carries a positive charge of 0.35 *e*. The charge distribution of Zn-MoS_2_ can be confirmed from Figure 1B. It is easy to see that Zn atoms are surrounded by cyan, which indicates that Zn atoms lose electrons. In addition, the magnetic moment of MoS_2_ is produced by Zn doping, the total magnetic moment of the single-layer MoS_2_ is 2.00 *µ*_B_, only 0.1*µ*_B_ is located on the doped Zn atom, which indicates that the magnetic moment of the system mainly comes from Mo atom. In order to further understand the electronic and magnetic properties of Zn-MoS_2_, the total DOS (TDOS) and projected DOS (PDOS) of Zn-MoS_2_ spin polarization are given in Figure 1C. It can be seen from Figure 1C that an asymmetric DOS peak appears near the Fermi level, which is obviously different from the perfect MoS_2_ monolayer. This is consistent with the fact that the Zn-MoS_2_ system is paramagnetic (2.0 *µ*b). Compared with MoS_2_ monolayer Supplementary Figure S1B, the spin down channel of Zn-MoS_2_ maintains the zero gap semiconductor characteristics of MoS_2_, but the spin up channel presents a non-zero density of states near Fermi level which indicates that the Zn-MoS_2_ system is semimetallic. Seen from the PDOS shown in Figure 1C, the degree of spin asymmetry of the 4*d* orbit of Mo is greater than that of the 3*d* orbit of Zn. This is consistent with the fact that the magnetic moment is mainly located on Mo atoms near Zn doping. In addition, since the asymmetric DOS peak is dominated by the 4*d* orbit of Mo, it can be expected that the spin charge density is mainly distributed on Mo atoms. Therefore, as shown in Figure 1B, the spin charge density of Zn-MoS_2_ system is mainly concentrated around Mo atom. In addition, Figure 1C also shows that near the Fermi level, the 3*p* state of Zn atom is hybridized with the 4*d* orbital of Mo, indicating the interaction between metal atom and S-vacancy.

### Adsorption of H_2_CO on Original MoS_2_ Monolayer

**FIGURE 2:**
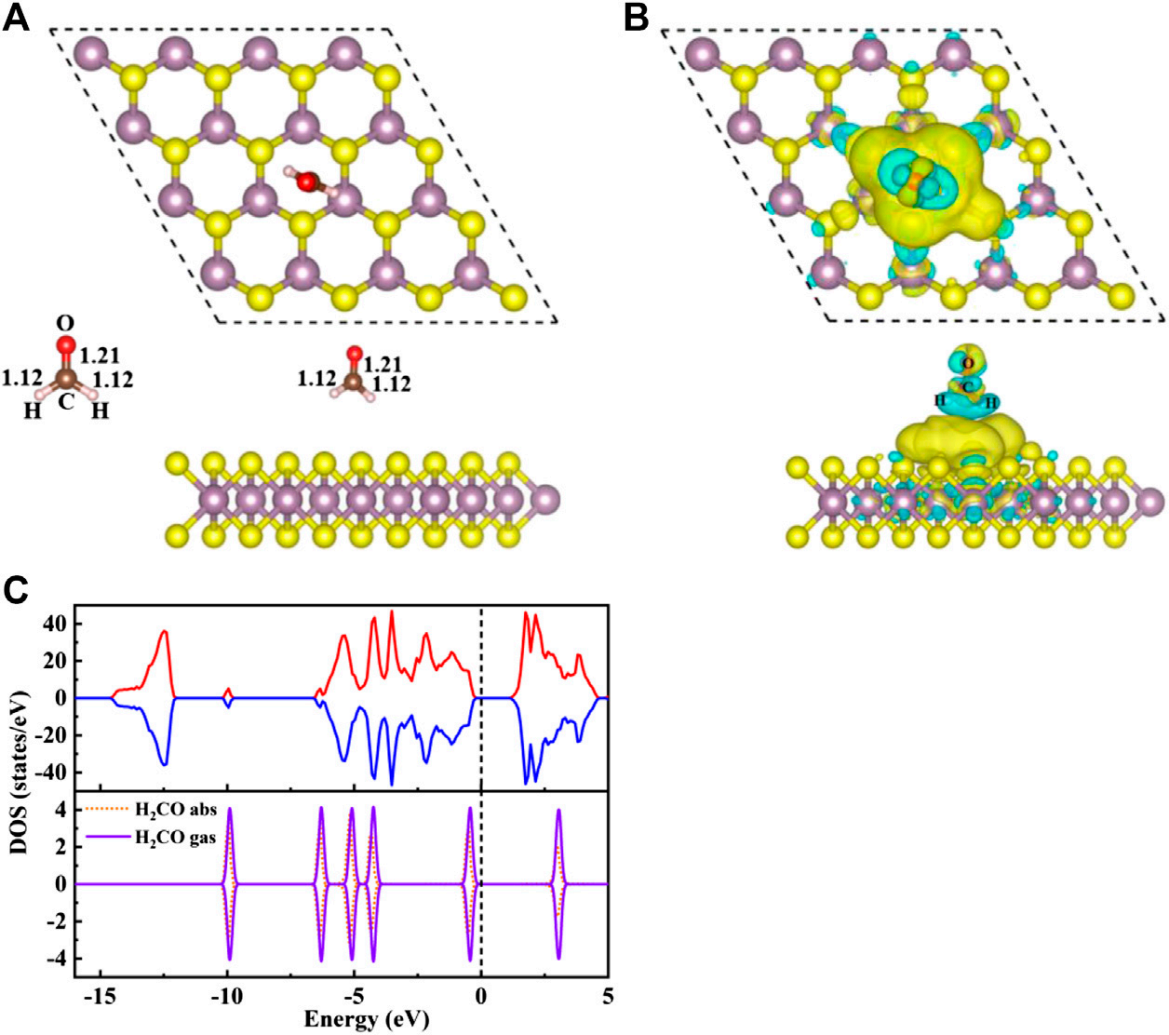
**(A)** The top view **(top)** and side view **(bottom)** structure of S/H_2_CO, and the left side shows the structure of H_2_CO in the gas phase. **(B)** Charge density difference diagram of S/H_2_CO. The yellow area indicates charge accumulation and the cyan area indicates charge loss. **(C)** The spin polarized TDOS of S/H_2_CO **(upper panels)** and the projected density of states (PDOS) of the original monolayer MoS_2_ for H_2_CO adsorption **(lower panels)**: Orange dotted line, H_2_CO in the adsorption state; purple curve, H_2_CO in the gas phase. The positive and negative values of DOS indicate spin up and spin down, respectively. The Fermi level is marked with a black dotted line.

In order to obtain a stable configuration, various possible initial adsorption structures were considered Supplementary Figure S1A. The interaction between H_2_CO and original MoS_2_ is very weak, and the stable configuration obtained belongs to physical adsorption. In this work, only the configuration with the largest and most stable adsorption energy is discussed. The adsorption energy, charge transfer and other related parameters are shown in Table 1, and the geometric electronic structure is shown in Figure 2.

**TABLE 1:** Parameters of stable configuration of H_2_CO adsorbed on original and Zn doped monolayer MoS_2_: adsorption energy (*E*_ad_ in eV), charge obtained by H_2_CO (*Q*_g_ in *e*), charge obtained by Zn (*Q*_Zn_ in e), magnetic moment of Zn atom (*M*_Zn_ in *µ*_B_), nearest distance between adsorbed molecule and Zn atom (*d*_g-Zn_ in Å), average distance between Zn atom and its adjacent molybdenum atom (*d*_s/Zn-Mo_ in Å), The height of Zn atom relative to S plane (*h* in Å).

Configuration	*E*_ad_	*Q*_g_	*Q*_Zn_	*M*	*M*_Zn_	*d*_g-Zn_	*D*_s/Zn-Mo_	*h*
S/H_2_CO	0.04	−0.01	0.00	0.00 (0.00)	0.00	3.13	2.41	0.00
Zn/H_2_CO-(a)	0.98	−0.05	0.51	1.98 (0.05)	0.08	1.98	2.70	0.33
Zn/H_2_CO-(B)	0.80	−0.09	0.53	1.97 (0.05)	0.08	1.95	2.70	0.28
Zn/H_2_CO-(c)	0.15	0.07	0.33	2.00 (−0.01)	0.09	2.02	2.67	0.27
Zn/H_2_CO-(d)	0.11	0.08	0.33	2.00 (−0.01)	0.10	2.02	2.66	0.25

**FIGURE 3:**
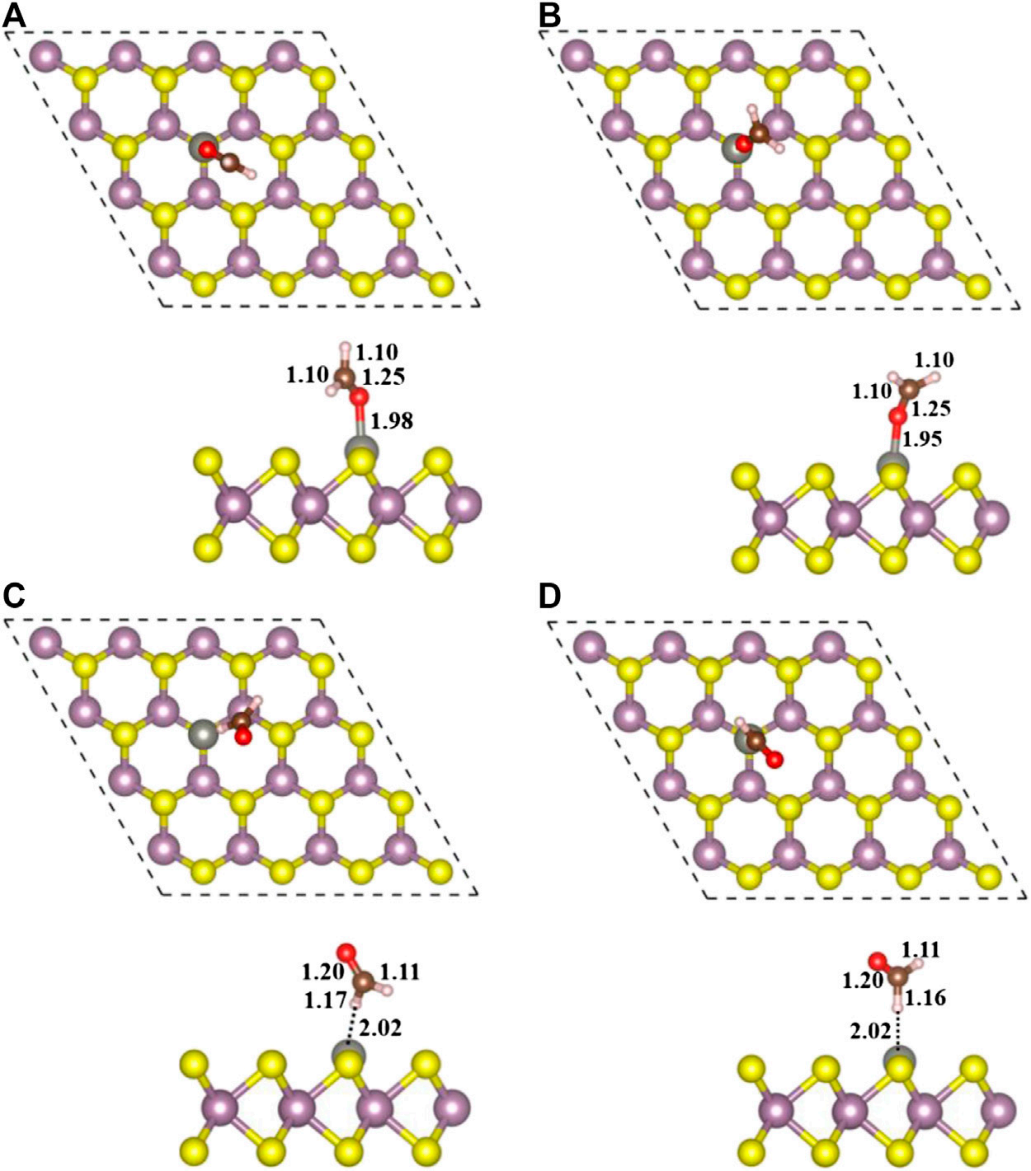
Top view **(top)** and side view **(bottom)** of geometric structures of Zn/H_2_CO-**(A)**, Zn/H_2_CO-**(B)**, Zn/H_2_CO-**(C)** and Zn/H_2_CO-**(D)** configurations are shown in **(A–D)**, respectively.

For M (*μ*_B_), the values inside and outside the brackets are the magnetic moment of the adsorbed molecule and the magnetic moment of the whole cell, respectively. It should be noted that S/H_2_CO refers to the configuration shown in Figure 3A, and the dopant of this configuration is actually S atom.

Seen from Figure 2A, the adsorption energy of H_2_CO on the original monolayer MoS_2_ is 0.04 eV. The adsorption of H_2_CO is almost perpendicular to the plane, and the H-end is downward. The nearest distance between the molecule and the substrate is 3.13 Å. Due to the small adsorption energy, the interaction between the adsorbed molecules and the substrate is weak, and the geometric structure of the adsorbed molecules is almost undisturbed. The C-O bond length is about 1.21 Å, and C-H bond length is about 1.12 Å, which is the same as that of H_2_CO bond in gas phase Figure 2A. The position of S atom under the adsorbed H_2_CO does not change, and the bond length between S and Mo is still 2.41 Å. This result can also be confirmed by Figure 2B where H_2_CO is almost surrounded by cyan, and only 0.01 e was obtained from the original monolayer MoS_2_. In order to further understand the interaction between the adsorbed H_2_CO and the original monolayer MoS_2_, we also calculated the TDOS of S/H_2_CO structure and the DOS of H_2_CO before and after adsorption, as shown in Figure 2C. After the adsorption of H_2_CO, we can see that there is no induced impurity state, and the band gap has no obvious change. Just because of the introduction of molecules, the single peak increases, indicating that the adsorption almost does not change the electrical properties of the original monolayer MoS_2_, It is well consistent with the literature report (Ma et al., 2016b). In other words, the original monolayer MoS_2_ is not sensitive to H_2_CO, which further proves that the adsorption of H_2_CO on the original monolayer MoS_2_ belongs to physical adsorption.

### Adsorption of H_2_CO on Zn Doped Monolayer MoS_2_ (Zn/H_2_CO)

Finally, four stable configurations of H_2_CO adsorption structure on Zn-MoS_2_ were obtained, including Zn/H_2_CO-(a), Zn/H_2_CO-(b), Zn/H_2_CO-(c) and Zn/H_2_CO-(d). The structure parameters are shown in Figure 3, and the other related parameters are shown in Table 1.

#### Configuration of the Adsorbed Zn/H_2_CO-(a) and Zn/H_2_CO-(b)

As shown in Figure 3A,B, the adsorption energies of Zn/H_2_CO-(a) and Zn/H_2_CO-(b) are 0.98 and 0.80 eV, respectively. The distance between the O atoms of the adsorbed molecule and Zn dopant is 1.98 and 1.95 Å, respectively, indicating that the molecule has strong adsorbed ability. The interaction between the adsorbed H_2_CO and the substrate makes the geometry of H_2_CO change obviously, the C-O bond length of H_2_CO increased by 0.04 Å and the C-H bond length decreased by 0.02 Å. The average bond length of Zn-Mo on the substrate Zn-MoS_2_ increases from 2.41 to 2.70 Å, and the distance between the dopant Zn and the S-plane is 0.33 and 0.28 Å larger than that of the substrate Zn-MoS_2_, respectively. In addition, Bader charge analysis showed that H_2_CO was the electron acceptor of Zn/H_2_CO-(a) and Zn/H_2_CO-(b), and the electron transfer between Zn-MoS_2_ substrate and H_2_CO was 0.05 and 0.09 *e*, respectively, this is mainly due to the doping of Zn atoms (0.51 and 0.53 *e*), it may be due to the difference of electronegativity between O (3.44) and Zn (1.65).

**FIGURE 4:**
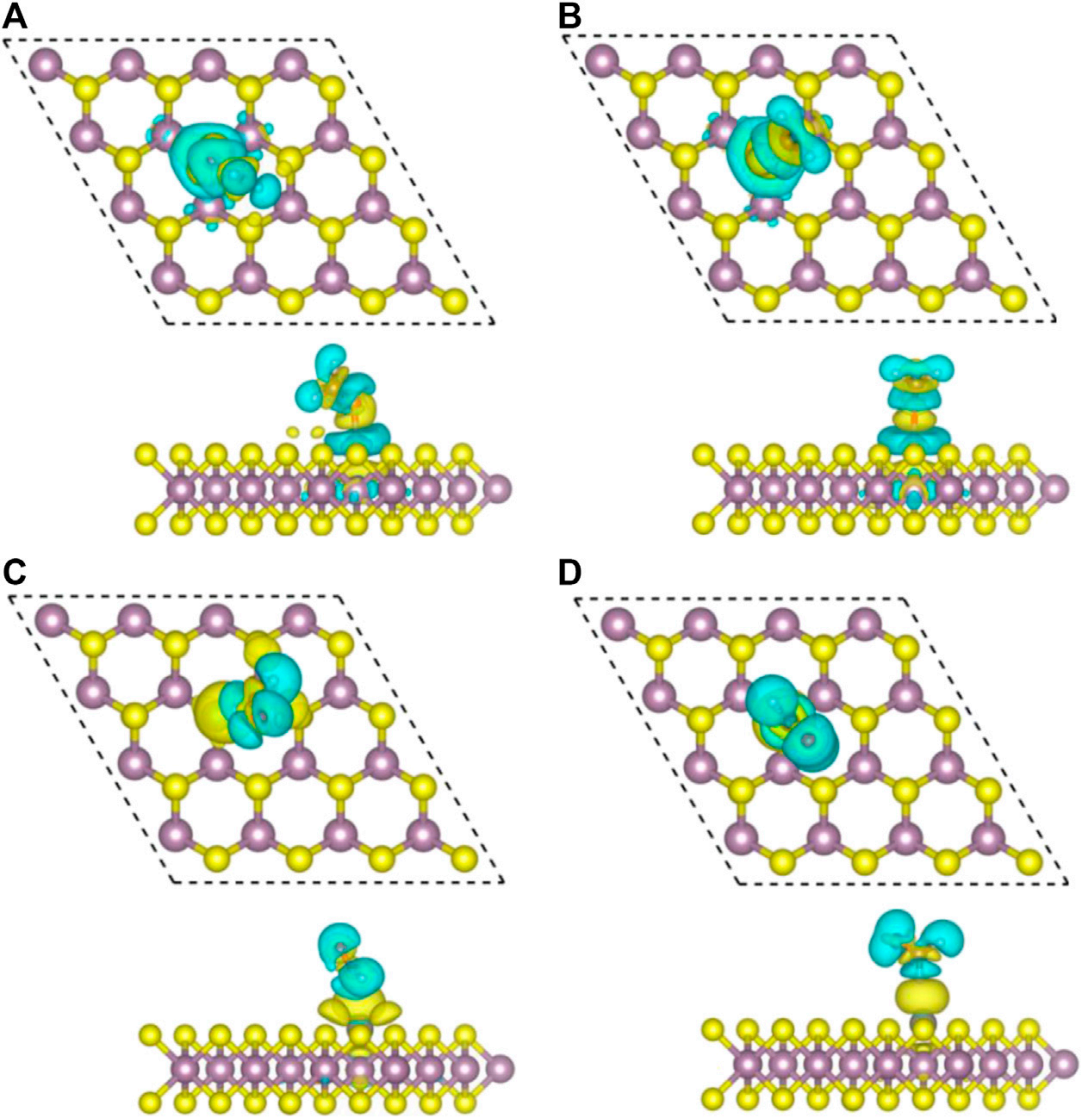
Top view **(top)** and side view **(bottom)** of charge density differences of final configurations of Zn/H_2_CO-**(A)**, Zn/H_2_CO-**(B)**, Zn/H_2_CO-**(C)** and Zn/H_2_CO-**(D)** are shown in **(A–D)**, respectively. Yellow and cyan represent regions of electron accumulation and depletion, respectively.

The difference of charge density of the final configuration of Zn/H_2_CO-(a) and Zn/H_2_CO-(b) is shown in Figures 4A,B to further understand the interaction between H_2_CO molecule and Zn doped monolayer MoS_2_. The yellow area is the electron accumulation area, and the cyan area is the electron consumption area. As shown in Figure 4A, the electron transfer is not only located on the C and O atoms of H_2_CO adsorption, but also on the O-Zn bond, which is consistent with the strong adsorption capacity of H_2_CO. In addition, the loss of electrons on the C-O bond leads to the weakening of the C-O bond, which makes the O atom protruding above the S plane chemically active to other molecules, including H_2_CO itself. For the adsorption of Zn/H_2_CO-(b), it can be found that the adsorption behavior is similar to that of Zn/H_2_CO-(a), as shown in Figure 4B, which will not be further discussed.

**FIGURE 5:**
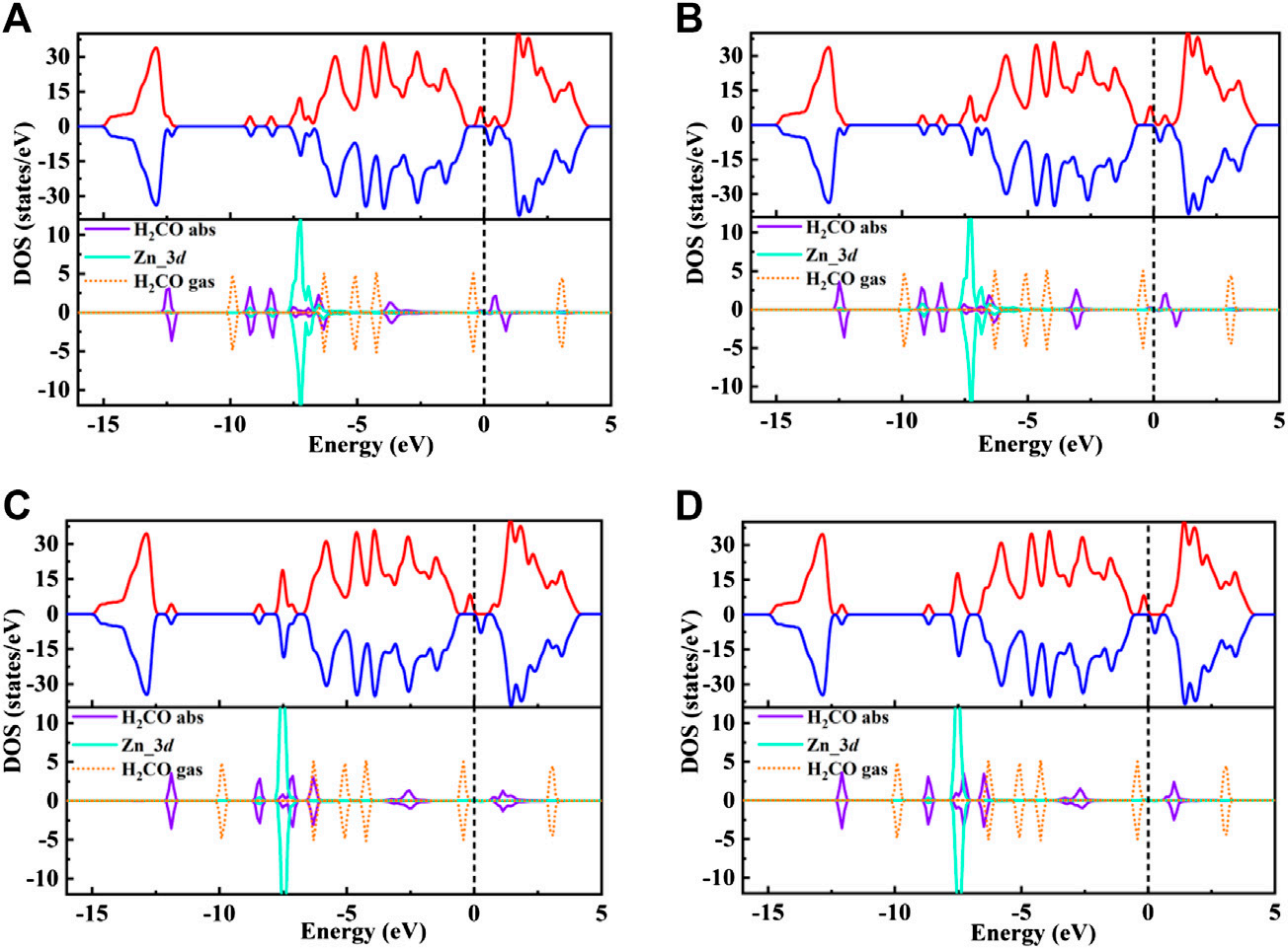
**(A**–**D)** shows the total spin polarized density of states (TDOS) of Zn/H_2_CO-**(A)**, Zn/H_2_CO-**(B)**, Zn/H_2_CO-**(C)** and Zn/H_2_CO-**(D)** systems **(upper panels)**, and the projected density of states (PDOS) of H_2_CO adsorbed by Zn-MoS_2_ **(lower panels)**: Purple curve, H_2_CO in adsorption state; orange dotted line, H_2_CO in gas phase; blue curve, *d*-projected PDOS for Zn atom. The positive and negative values of DOS indicate spin up and spin down, respectively. The Fermi level is marked with a black dotted line.

In order to further understand the adsorption behavior of H_2_CO on Zn-MoS_2_ surface. Figures 5A–D displayed the spin-polarized total densities of states (TDOS) (upper panels) and corresponding DOS projected on 3*d* states of Zn atom, adsorbed H_2_CO gas molecules and the isolated H_2_CO gas molecules (lower panels) after H_2_CO adsorption on Zn-embedded monolayer MoS_2_.

**FIGURE 6:**
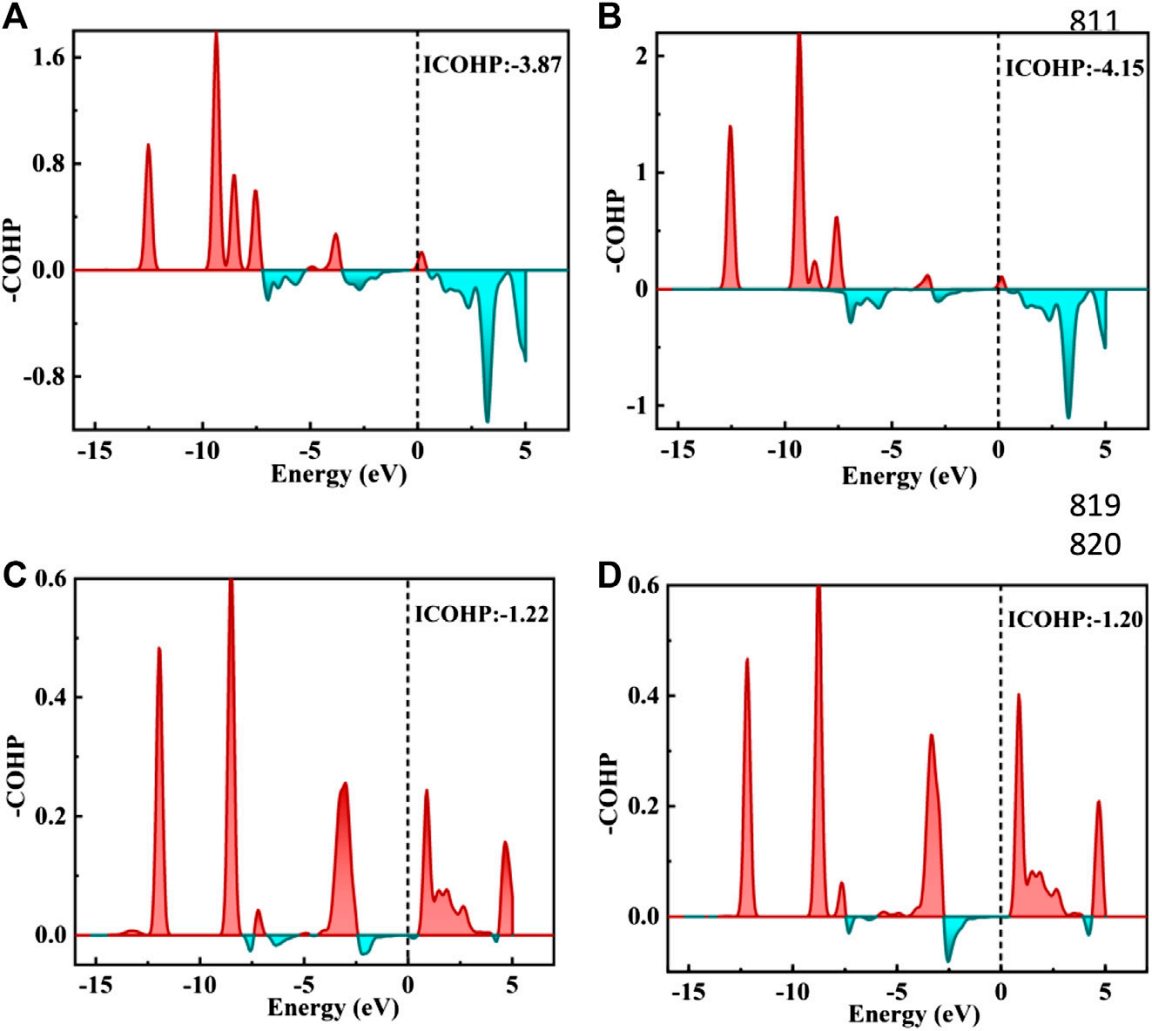
**(A–D)** figure shows the negative crystal orbital Hamilton density (-COHP) on the Zn/H_2_CO-**(A)** and Zn/H_2_CO-**(B)** system O-Zn bond and the Zn/H_2_CO-**(C)** and Zn/H_2_CO-**(D)** system H-Zn bond, respectively. The red filled area is the O-Zn/H-Zn bonding area, and the green filled area is the O-Zn/H-Zn antibonding. Fermi energy level marked with black dotted line.

The TDOS of Zn/H_2_CO-(a) and Zn/H_2_CO-(b) systems are shown in Figures 5A,B. Compared with Zn-MoS_2_, due to the hybridization of Zn atoms and H_2_CO molecules, the charge is transferred from the matrix to the adsorbed H_2_CO molecule, resulting in a new DOS peak at CBM for the TDOS of Zn/H_2_CO-(a) and Zn/H_2_CO-(b) systems. It can be observed that the occupied state PDOS of the adsorbed H_2_CO molecule Figures 5A,B, below is much lower panels than Fermi level, and the induced impurity state is produced, which reveals the reaction between Zn-MoS_2_ monolayer and H_2_CO molecule. For Zn/H_2_CO-(a) and Zn/H_2_CO-(b) configurations, the molecular orbitals of adsorbed H_2_CO are delocalized relative to the isolated H_2_CO in the gas phase. The 3*d* orbital of Zn atom is coupled with H_2_CO in the range of 9.60∼0.00 eV. The interaction between H_2_CO molecule and Zn atom leads to charge transfer. Zn/H_2_CO-(a) and Zn/H_2_CO-(b) structures have higher adsorption energy, which indicates that the adsorption is chemisorption, which is suitable for gas removal under H_2_CO adsorption. The results show that, compared with undoped MoS_2_, doping Zn atoms in the defective monolayer MoS_2_ is beneficial to the adsorption of H_2_CO. We use crystal orbital Hamiltonian populations (COHP) to analyze the interaction between different O-Zn/H-Zn bonds by means of a pair of atomic orbital bonding or antibonding states (van Santen and Tranca, 2016; Steinberg and Dronskowski, 2018; Tsuji and Yoshizawa, 2018). Moreover, through the integration of COHP (ICOHP), we can get a precise value of bond strength. The positive and negative values of ICOHP represent the anti bonding state and bonding state respectively. The smaller the ICOHP value, the stronger the bond strength. Figures 6A-D shows the -COHP curve and the integral (ICOHP) value of the O-Zn and H-Zn bonds in the structured Zn/H_2_CO-(a) and Zn/H_2_CO-(b) systems and the Zn/H_2_CO-(c) and Zn/H_2_CO-(d) systems, respectively. It can be seen from Figures 6A,B that when H_2_CO is adsorbed to Zn-MoS_2_, there are a small amount of O-Zn bonding orbitals (-COHP values) at Fermi level, and the orbitals above Fermi level belong to anti bonding orbitals. In other words, the O-Zn bond is enhanced after H_2_CO adsorption, which is consistent with the increase of C-O distance (Figures 4A,B). The O atoms in the two adsorption systems have strong binding with Zn atoms (ICOHP value is small), which also shows that these phases are stable.

#### Configuration Adsorption of Zn/H_2_CO-(c) and Zn/H_2_CO-(d)

As shown in Figures 3C,D the Zn/H_2_CO-(c) and Zn/H_2_CO-(d) configurations with adsorption energies of 0.15 and 0.11 eV, respectively, are much less stable than those of Zn/H_2_CO-(a) and Zn/H_2_CO-(b), and the H atoms point to the doped Zn atoms. Different from the configurations of Zn/H_2_CO-(a) and Zn/H_2_CO-(b), the adsorption energies are also very different, similar to the adsorption on the original monolayer MoS_2_. Therefore, the orientation of H atom or O atom in H_2_CO can be used to determine the adsorption energy. At the same time, in Figures 3C,D the interaction between the adsorbed molecules and the substrate is also different due to the different orientations of hydrogen atoms, i.e., the angle of C-H-Zn is different. In the two structures, the distance between H and Zn atoms is 2.02 Å.

Therefore, the strong covalent bond or ionic bond between the two atoms can be eliminated. The weak electrostatic attraction of negatively charged H atom (0.06 *e*) and positively charged Zn atom (0.33 *e*) should be combined, which is consistent with the weak adsorption of Zn/H_2_CO-(c) and Zn/H_2_CO-(d) configurations.

Compared with H_2_CO in the gas phase, the geometry and substrate of the adsorbed state are only slightly changed. The C-O bond of H_2_CO decreased by 0.01 Å. In addition, for Zn/H_2_CO-(c) and Zn/H_2_CO-(d), there is a small electron transfer (0.07 and 0.08 *e*) between the adsorbed H_2_CO and the substrate, and the molecule has a small magnetic moment of 0.01 µ_B_. The adsorption of Zn/H_2_CO-(c) and Zn/H_2_CO-(d) is weaker than that of Zn/H_2_CO-(a) and Zn/H_2_CO-(b), indicating that the probability of occurrence of the latter two configurations is much higher than that of the former two. For the adsorption of Zn/H_2_CO-(c) and Zn/H_2_CO-(d) configurations, the charge density difference diagram shows that there is almost no electron accumulation between the adsorbed molecule H_2_CO and the Zn doped MoS_2_ monolayer (Figures 4C,D). It is further confirmed that the adsorption capacity of H_2_CO on the substrate is weak, which is consistent with the small adsorption energy and large distance mentioned above.

Further analyzing the adsorption behavior of H_2_CO on the Zn-MoS_2_ surface, it can be seen from Figures 5C,D above that the TDOS of the Zn/H_2_CO-(c) and Zn/H_2_CO-(d) systems do not show induced impurity state near the Fermi level, and the DOS curves of the adsorbed H_2_CO of the Zn/H_2_CO-(c) and Zn/H_2_CO-(d) systems overlap slightly with the 3*d* orbitals of the Zn atoms (Figures 5C,D, below), shows that the interaction between H_2_CO and Zn-MoS_2_ is weak, the bonding between H-Zn atoms is weak, and the ICOHP negative value is large (Figures 6C,D), resulting in only 0.07 and 0.08 *e* being transferred from the adsorbed H_2_CO molecule to the final substance. The H-Zn strength in Zn/H_2_CO-(c) system is higher than that in Zn/H_2_CO-(d) system, indicating that Zn/H_2_CO-(c) system is more stable than Zn/H_2_CO-(d) system. This conclusion is consistent with the previous calculation of adsorption energy (Table 1). In terms of magnetic properties, the total magnetic moment of the whole adsorption system did not change after adsorption of H_2_CO by Zn-MoS_2_ (Zn/H_2_CO-(c) and Zn/H_2_CO-(d) were 2.00 *µ*_B_), which was consistent with the small adsorption energy of Zn/H_2_CO-(c) and Zn/H_2_CO-(d).

## Conclusion

In conclusion, according to the first principles calculations, we have studied the effects of Zn doping S vacancy on the electronic structure, magnetic properties and chemical activity of monolayer MoS_2_. The calculation of binding energy shows that Zn atoms are closely bound to S-defects, which is mainly due to the hybridization between dopant atoms and their nearest Mo atoms. Finally, a new single cluster catalyst on ZnMo_3_ surface is formed, which improves the selectivity of MoS_2_ surface. By embedding Zn atoms, the magnetic properties of MoS_2_ monolayer can be adjusted and the spin magnetic moment of Zn-MoS_2_ is 2.00 *µ*_B_. The electronic properties of MoS_2_ are also changed by the impurity states induced in the band gap. The effects of H_2_CO adsorption and doping on the chemical activity of MoS_2_ monolayers were further investigated. It was found that the H-end downward adsorption of H_2_CO in the original monolayer MoS_2_ and Zn doped monolayer MoS_2_ was very weak, and the electronic structure of the two substrates changed little after adsorption, indicating that the two systems were not sensitive to H_2_CO.When the O atom in H_2_CO molecule faces to the substrate, the adsorption capacity is strong, and the adsorbed H_2_CO is effectively activated. DOS analysis showed that the electronic structure of Zn-MoS_2_ could be changed by introducing impurities in the band gap when the O-terminal of H_2_CO molecule was adsorbed downward. At the same time, the magnetic properties of Zn-MoS_2_ system are also adjusted. The COHP diagram shows that Zn atoms are strongly bonded with O atoms. This study shows that Zn doping is a promising method to optimize the electronic structure, magnetic properties and chemical activity of MoS_2_, which provides a promising way to improve the electronic properties of MoS_2_ materials.

## Data Availability

The original contributions presented in the study are included in the article/Supplementary Material, further inquiries can be directed to the corresponding authors.
