# Supplementary material for: Formaldehyde Molecules Adsorption on Zn Doped Monolayer MoS2: A First-Principles Calculation
Source: Front Chem. 2021 Apr 16;8:605311. doi: 10.3389/fchem.2020.605311 (PMC8085485; doi:10.3389/fchem.2020.605311)
Supplement: Supplementary file 1 [file datasheet1.docx]

Formaldehyde molecules adsorption on Zn doped monolayer MoS_2_: A first-principles calculation

Huili Li ^1,†^, Ling Fu ^2,3†^, Chaozheng He ^4,5 ^[[1]](#footnote-2)^*^, Jinrong Huo ^4^, Houyong Yang ^4,5^, Tingyue Xie ^6^, Guozheng Zhao ^1 *^, Guohui Dong ^7 *^

*^1^ Key Laboratory of Magnetic Molecules, Magnetic Information Materials Ministry of Education, The School of Chemistry and Material Science, Shanxi Normal University, Linfen 041004, PR China*

*^2^ College of Agricultural Engineering, Nanyang Normal University, Nanyang, Henan 473061, China*

*^3^ College of resources and environmental engineering, Tianshui Normal University, Tianshui 741001, China*

*^4^ Institute of Environmental and Energy Catalysis, School of Materials Science and Chemical Engineering, Xi’an Technological University, Xi’an 710021, China*

*^5^ Shaanxi Key Laboratory of Optoelectronic Functional Materials and Devices, School of Materials Science and Chemical Engineering, Xi'an Technological University, Xi'an 710021, China*

*^6^ School of Physics and Electronic Science, Shanxi Datong University, Shanxi 037009, P. R. China*

*^7^ School of Environmental Science and Engineering Shaanxi University of Science and Technology, Xi’an 710021, China*

**(b)**




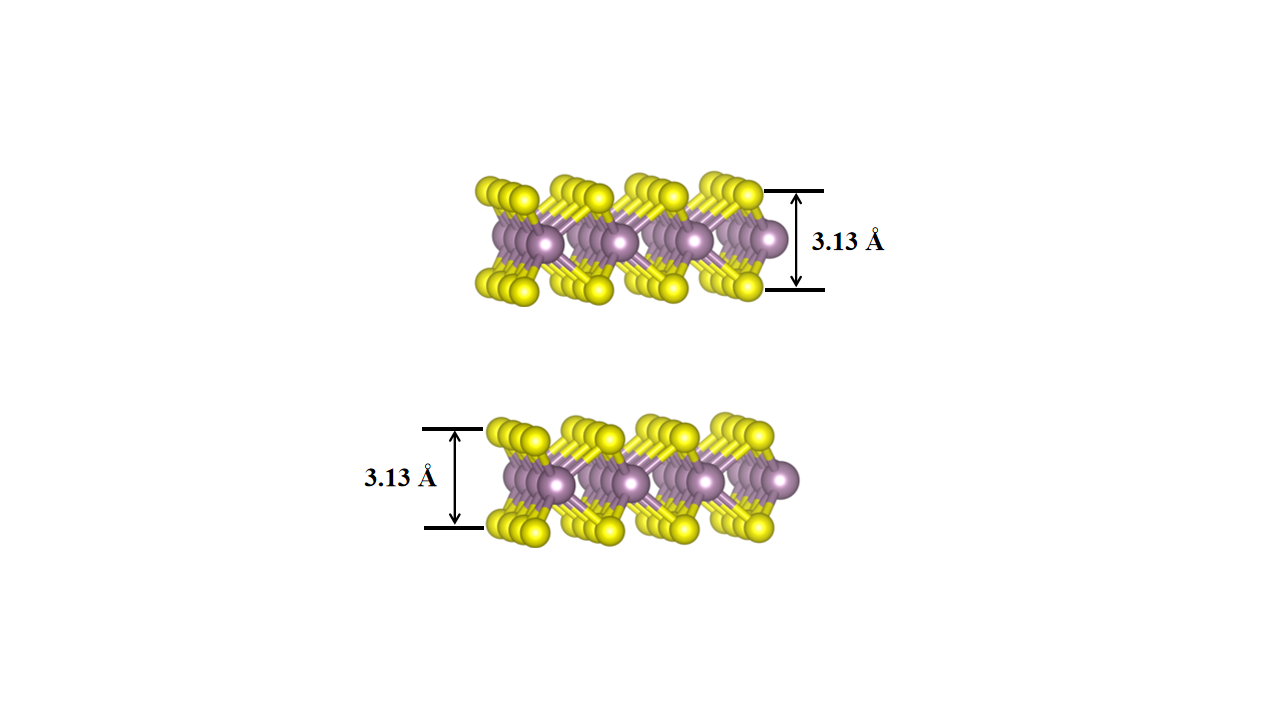


**(a)**


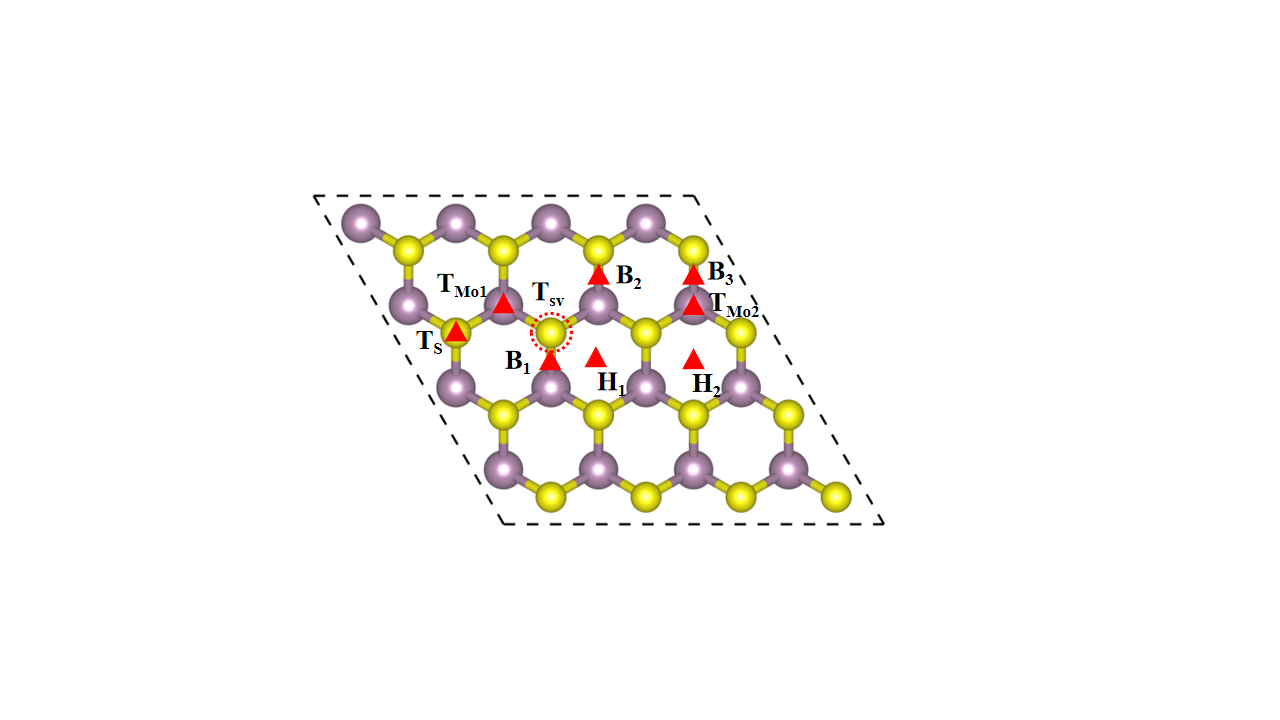


Fig.S1 (a) top view (top) and side view (bottom) of 4 × 4 supercell monolayer MoS_2_ structure. The possible sites of H_2_CO adsorption by monolayer MoS_2_ are shown on the top. S atoms and Mo atoms are yellow (small) and lavender (large) spheres, respectively. (b) The spin polarized band structure (left) and total density of states (right) of monolayer MoS_2_ are studied. The red (blue) line represents the spin up (spin down) band, respectively. The Fermi level is marked with a black dotted line.

1. * Corresponding authors. E-mail: [hecz2019@xatu.edu.cn](mailto:hecz2019@xatu.edu.cn) (C. He); zhaoguozheng@sxnu.edu.cn (G. Zhao); dongguohui@sust.edu.cn (G. Dong).

   ^†^ These authors contributed equally to this work. [↑](#footnote-ref-2)
